# Supplementary material for: A Brief Online Implicit Bias Intervention for School Mental Health Clinicians
Source: Int J Environ Res Public Health. 2022 Jan 7;19(2):679. doi: 10.3390/ijerph19020679 (PMC8776032; doi:10.3390/ijerph19020679)
Supplement: Supplementary file 1 [file ijerph-19-00679-s001.zip › ijerph-1466776-supplementary.pdf]

Implicit Bias Knowledge Quiz

Answers in **bold**

1. Stereotyping is…
    a. an automatic cognitive processes that often operates beyond our conscious awareness
    b. often inaccurate and can contribute to biased decision-making
    c. a crucial survival skill emerging from eons of human evolution
    d. All of the above

2. Stereotypes can operate and affect our behaviors…
    a. as long as we can access the stereotype information in our memory
    b. even when we do not believe them to be true
    c. even if we gain awareness and use effective strategies to counter them
    d. a & b

3. Implicit bias can affect our behavior and interactions with others…
    a. only when we are aware of the biases
    b. despite our intentions to be egalitarian
    c. when we are with others like us (in-group members)
    d. only when we feel threatened

4. Clinicians' implicit bias contributes to…
    a. Poor treatment outcomes for minority youth (i.e., health disparities)
    b. Strained clinician-student interactions and weak therapeutic alliance
    c. Poor student treatment engagement and premature dropout
    d. All of the above

5. The "Bias Blind Spot" refers to…
    a. The inability to see oneself as the victim of bias.
    b. The difficulty with recognizing bias operating in one's own mind.
    c. The inability of the dominate group to see bias all around them.
    d. The difficulty of seeing one's significant other as biased against us.

6. The Implicit Association Test…
    a. Is a research tool designed to assess one's implicit bias based on reaction times
    b. is designed to identify racist ideology
    c. detects one's unconscious associations with biased or egalitarian beliefs
    d. is a very accurate test of one's true beliefs

7. Which of the following is an effective implicit bias neutralization strategy (select all that apply)
   a. **Finding out what you have in common with someone**
   b. Enthusiastically asserting egalitarian beliefs
   c. **Actively seeking out information to challenge your assumptions**
   d. Systematically identify how implicit biases is operating in everyone around you

8. Seeking Commonality can be an effective bias neutralization strategy… (select all that apply)
   a. **especially when used with open-ended questions to tap into core values.**
   b. because shared interests can foster mutual attraction and friendship
   c. only when both parties have a lot in common.
   d. **because learning that you have some things in common with someone can help you see them more like an in-group member (e.g., one of us).**

9. Which of the following is not an effective approach for gaining perspective to better understand someone else's experience:
   a. **Carefully imagine how you would feel and what you would do in that person's situation**
   b. Take into consideration the person's context, history, and lived experiences, before imagining how *they* would feel in their situation.
   c. Be genuinely curious about their experiences and listen with an open mind.
   d. Accept the reality of their experience even if it challenges some of your own beliefs.

10. Confirmation Bias is…
    a. The tendency to believe that everything positive is true
    b. **The tendency for the brain to seek out and retain information that supports one's beliefs, while ignoring/dismissing information that challenges our beliefs.**
    c. Harmless because everyone is prone to it, thereby equaling the playing field
    d. Easy to challenge because the brain can switch to attending to belief-inconsistent information if one simply shifts their intentions.
